# Disability-adjusted life years (DALYs) based COVID-19 health impact assessment: a systematic review

**DOI:** 10.1186/s12889-023-15239-0

**Published:** 2023-02-15

**Authors:** Daniel Teshome Gebeyehu, Leah East, Stuart Wark, Md Shahidul Islam

**Affiliations:** 1 School of Health, Faculty of Medicine and Health, University of New England, Armidale, NSW 2351 Australia; 2 School of Veterinary Medicine, Wollo University, 1145 Dessie, Amhara Ethiopia; 3 School of Nursing and Midwifery, Health, Engineering & Sciences, University of Southern Queensland, Ipswich, Queensland 4305 Australia; 4 School of Rural Medicine, Faculty of Medicine and Health, University of New England, Armidale, NSW 2351 Australia

**Keywords:** COVID-19, Health, Impact, Disability-adjusted life years, Systematic review

## Abstract

**Background:**

The emergence of COVID-19 has resulted in health, socio-economic, and political crises. The overall health impact of this disease can be measured by disability-adjusted life years (DALYs) which is the sum of the life years lost due to disability (YLDs) and the years life lost due to premature death (YLLs). The overarching objective of this systematic review was to identify the health burdens of COVID-19 and summarise the literature that can aid health regulators to make evidence-based decisions on COVID-19 mitigation strategies.

**Methods:**

This systematic review was conducted using the PRISMA 2020 guidelines. DALYs-based primary studies were collected from databases, manual searches, and included studies’ references. The primary studies published in English language, conducted since the emergence of COVID-19, and using DALYs or its subsets (years life lost due to disability and/or years life lost due to premature death) as health impact metrics, were the inclusion criteria. The combined disability and mortality health impact of COVID-19 was measured in DALYs. The risk of bias due to literature selection, identification, and reporting processes was assessed using the Joanna Bridges Institute critical appraisal tool for cross-sectional studies, and the certainty of evidence was assessed using the GRADE Pro tool.

**Result:**

Of the 1459 identified studies, twelve of them were eligible for inclusion in the review. The years life lost due to COVID-19 related mortality was dominant over the years life lost due to COVID-19 related disability (disability times from the onset of COVID-19 to recovery, from diseases occurrence to mortality, and the long-term consequences of COVID-19) in all included studies. The long-term consequence disability time and the pre-death disability time were not assessed by most of the reviewed articles.

**Conclusion:**

The impact of COVID-19 on both the length and quality of life has been substantial and has been causing considerable health crises worldwide. The health burden of COVID-19 was greater than other infectious diseases. Further studies focussing on issues examining increasing preparedness for future pandemics, public sensitization, and multi-sectorial integration are recommended.

## Introduction

Coronavirus disease 2019 (COVID-19) is a respiratory infectious disease caused by severe acute respiratory syndrome coronavirus two (SARS-COV-2) and it was named “COVID-19” by the World Health Organization (WHO) on 11 February 2020. COVID-19 was identified as a global public health threat and nominated as a pandemic on 30 January and 11 March 2020 respectively [1]. COVID-19 is causing substantial health, political, and socio-economic crises since its emergence [2–4]. Nearly half a billion cases and 6.2 million deaths are reported, two years post declaration as a pandemic [5]. COVID-19 is considered potentially the greatest public health threat since the emergence of pandemic influenza in 1918. If the fatality and morbidity of COVID-19 continue at this rate, it will compromise public health [6]. COVID-19 shows a wide variety of signs ranging from asymptomatic to severe symptoms; according to the Australian Government Department of Health [7] cough, tiredness, fever, and loss of taste and smell are the most common signs of COVID-19. It is swiftly contagious, and its fatality rate varies from 0.1 to 25% [8]. On average, COVID-19 has 14 days of severe illness and about 28 days of long-term consequences [9]. Regardless of their geographic location and economic status, COVID-19 is severely affecting individuals, sectors, countries, and global society in general. This is not due only to its direct health impacts, as individuals may also affected indirectly through measures and restrictions (lockdown, moment restriction, and job terminations) adopted for the prevention of COVID-19 pandemic.

### Rationale

Disability-adjusted life years (DALYs) is one of the leading quantitative health burden assessment techniques [10]. One DALY represents the loss of 1 year of healthy life. According to the WHO global health estimates [10], the impact of a condition, situation or disease arising from a particular cause is the sum of years of life lost due to premature mortality (YLLs) and years of life lost due to disability (YLDs). YLDs because of COVID-19 are calculated by considering the disability times (pre-mortality, pre-recovery, and long-term consequences times). Some studies [9] include the long-term consequences (tired, pain all over the body after acute infection, and fatigue) of COVID-19 in YLD calculation while others do not [11]. In addition, several studies did not consider the pre-mortality illness time in YLD calculation [9, 11–13] and they only considered the YLLs of deceased without including the disability time from the onset of COVID-19 to death. Other studies [12, 14–16] had excluded the YLDs from DALYs calculation. Failing to include one or more of the disability times and/or total omission of YLDs from DALYs calculation can significantly compromise any attempt to quantify the health impact assessment of COVID-19. The health burden of COVID-19 is assessed on the quality of life (YLD), the length of life (YLL), or both. To assist both current and future health decision-makers in planning through health resources allocation and disease prioritisation, it is considered that summarizing the burden of COVID-19 using DALYs metrics is an important first step. Therefore, the overarching objective of this systematic review was to identify the health burdens of COVID-19 and produce a summary of the literature to aid health regulators in making evidence-based decisions on both current and future COVID-19 mitigations.

## Methodology

The Preferred Reporting Items for Systematic Reviews and Meta-Analyses (PRISMA) 2020 guidelines were used to prepare this systematic review. Before beginning this systematic review, a review protocol was prepared and registered in International Prospective Register of Systematic Reviews (PROSPERO) with a registration number of CRD42022324931. The protocol is published at PLOS ONE and available online at 10.1371/journal.pone.0274468.

### Eligibility criteria

Primary research articles on the impact of COVID-19 on health were the focus of this systematic review. Primary studies published from the date of COVID-19’s emergence (31 December 2019) to the end of literature search (25 April 2022) were included in the initial screen process, with any studies conducted using qualitative or quantitative techniques other than DALYs metrics then excluded. Primary research articles published in English language only were considered for review. The studies were included regardless of their consideration of long-term COVID-19 consequence (long COVID-19). The focus was on general population health, with any studies concentrating on a specific sub-group of the population (e.g., women, children, older people, individuals with intellectual disability etc) or on the specific health category (physical, mental, or social) not included.

### Information sources and search strategy

Three databases (Web of Science, Scopus, and PubMed) were searched during a period from 20 March to 25 April 2022. The publication dates (from 31 December 2019 to 25 April 2022), the publication language (English), and article type (primary studies) were used as filtering method to identify target articles. A combination of terms was used to identify eligible studies from databases. Since COVID-19 is known by different names, the search strategy was complex. As mentioned in the published protocol [17], the search term “*(Impact) OR (Burden) OR (Effect) AND (“COVID-19”) OR (“COVID 19″) OR (“SARS-COV-2″) OR (“SARS COV 2”) OR (“Coronavirus disease 2019”) OR (“Coronavirus disease-19″) OR (“Coronavirus diseases 19”) OR (“Severe acute respiratory syndrome coronavirus 2”) OR (“Severe acute respiratory syndrome coronavirus-2”) OR (“Wuhan coronavirus”) OR (Novel coronavirus 2019) AND (Health) AND (DALY) OR (DALYs) OR (“Disability-adjusted life years”)”* was used for the search. A snowball searching method was also used to access literature from Google Scholar, with any studies from the first 20 pages considered. The title (the impact of COVID-19 on human health) was also used to identify studies from Google Scholar. Following the initial identification of eligible papers, each of their references were then explicitly reviewed to attempt to recognise any further studies which were missed through the database searching processes.

### Selection and data collection processes

The first-round selection and screening processes were done by DTG. All the studies identified from databases, manual searches, and references of included studies were imported to EndNote X9 for further screening and de-duplication. Following the removal of duplicates, the non-relevant articles were manually removed using the title and abstract screening technique. Studies that were deemed eligible on that initial process were then further investigated using a full-text review and the articles that did not satisfy the eligibility criteria were removed. To ensure the quality and eligibility of articles and avoid missing valuable studies, the selection and data collection processes done by the lead author, DTG, was independently repeated by the three co-authors (MSI, LE, and SW). There were no disagreements among the authors.

### Data items

All papers regarding the negative health outcomes (quality of life, length of life and/or both) [18] due to COVID-19 were considered for analysis. It is expected to report the health burden in the form of YLDs when there was no mortality and in the form of YLLs when there was no recovery. The publication year, the objective of studies, the duration of data collections, the study coverage (country), the type of data collected (mortality, morbidity, or both), the study design, health impact metrics (YLDs, YLLs, or both/DALYs), and funding sources of the study were considered as data items for evidence synthesis.

### Study risk of bias assessment

The Joanna Briggs Institute (JBI) risk of bias assessment tool for cross-sectional studies was used. The JBI tool is formulated for assessing the risk of biases in reviewing cross-sectional primary studies with *Yes, No, Unclear or Not Applicable* answers.

### Effect measures and result synthesis

The effects of COVID-19 on the quality and length of life were measured using the disability-adjusted life years. DALYs due to COVID-19 were analysed using descriptive statistics (numerical figures, ranges, and percentages). The contribution of YLLs and YLDs to the total health burden (DALYs) was expressed using percentages in each study. From the DALYs subsets (YLDs or YLLs), the higher contributed to DALYs was identified for health intervention.

Even if COVID-19 is causing multi-dimensional crises, this systematic review is focused only on its health impacts. The synthesis of this review was concentrated on answering the following question:

A. How much the quality of life was affected by COVID-19? This question was answered using the YLDs arising from illness, distress, suffering and pain specifically associated with COVID-19.
B. How much the length of life was affected by COVID-19? This question was answered by calculating the shortened life expectancy (YLLs) due to COVID-19.

### Certainty assessment

The GRADE tool was used to assess the certainty of evidence from the included studies. This tool is designed to summarise the findings of the studies in the form of tables, with directness of findings, presence of publication biases, consistency of findings, study limitations, and inclusion of health outcomes (life quality and life length) considered as the factors for certainty of evidence. Based on the pre-set criteria (confounding and miss calculation effects) for GRADE modalities, the quality of evidence was judged as “*high, moderate, low, and very low.”* The certainty assessment was independently done by all the review authors and no disagreement occurred.

## Result

### Study selection

**Fig. 1:**
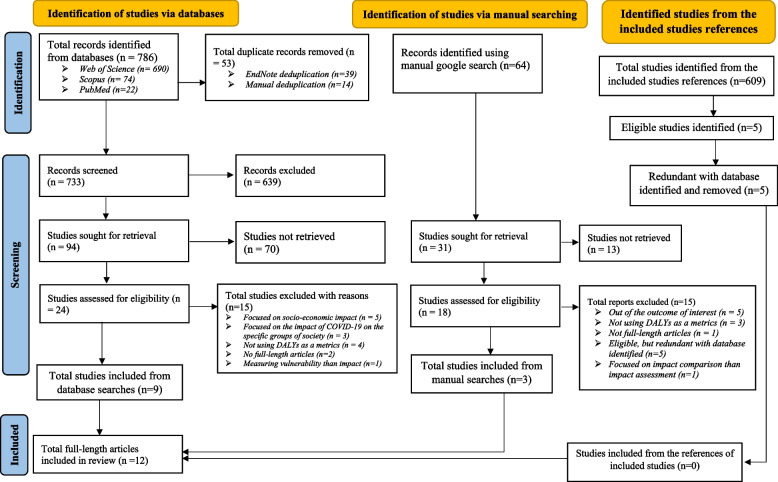
Study selection flow diagram (a model of PRISMA 2020)

A total of 1459 studies were initially identified in the databases (*n* = 786), manual Google scholar searches (*n* = 64), and from the references of included studies (*n* = 609) (Fig. 1). After detailed examination of these papers, a total of 12 eligible studies were included for the review. Nine [9, 11–13, 18–22] of the included studies were from the online databases and three [14–16] of them were from the manual Google scholar searches. The number of identified studies from three databases were from Web of Science (690), with just 74 from Scopus and 22 from PubMed (Fig. 1).

### Study characteristics

From the 12 included studies, seven [9, 11, 14, 15, 18–20] were published in 2021, and the other three [13, 16, 21] in 2022. Two studies [12, 22] were published in 2020. The aims of all studies were to assess or estimate the impact/burden/effect of COVID-19 on human health (Table 1) and used DALYs metrics to measure the health burden of COVID-19.

**Table 1:** The general characteristics of the reviewed studies

No.	Included studies	Objectives/aims of studies	Study duration	Country	Data	Data source	Study design	Impact metrics	Funding
1.	Cuschieri et al. [9]	“To estimate the direct impact of Covid-19 on the population”.	1 year	Malta	Mortality and morbidity	Mortality and weekly hospital reports	Observational	YLDs and YLLs	No funding received
2.	Fan et al. [11]	“To elucidate global disease burden with DALYs metric across different regions and periods.”	1 year and 3 months	Global	Mortality and morbidity	Open data reports	Observational	YLDs and YLLs	Ministry of science and technology, Taiwan
3.	Oh et al. [12]	“To estimate the YLLs due to COVID-19 in 30 high-incidence countries”.	3 months	30 high incidence countries	Mortality only	WHO daily reports	Observational	YLLs only	Ministry of health and welfare, republic of Korea
4.	Wyper et al. [13]	To estimate DALYs directly due to COVID-19 in Scotland, during 2020; and contextualise its population impact relative to other causes of disease and injury.	1 year	Scotland	Mortality and morbidity	National Records of Scotland	Observational	YLDs and YLLs	No funding received
5.	Islam et al. [14]	“To estimate the changes in life expectancy and years of life lost in 2020 associated with the covid-19 pandemic”.	1 year	37 countries	Mortality only	Online mortality database	Time series analysis	YLLs only	No funding received
6.	Pifarre et al. [15]	“To assess the years life lost due to COVID-19”.	9 months	81 highly affected countries	Mortality only	Weekly mortality data	Observational	YLLs only	La Caixa Foundation for Héctor Pifarré Arolas and Guillem López Casasnovas
7.	Ugarte et al. [16]	“To estimate the potential years of life lost in 17 countries”.	8 months	17 counties	Mortality only	Primary national source	Observational	YLLs only	The University of Nicosia Medical School
8.	Rommel et al. [18]	“To assess the health burden of COVID-19)”.	1 year	Germany	Mortality and morbidity	Report of Robert Koch Institute	Observational	YLDs and YLLs	Innovation Fund of the German Joint Federal Committee
9.	Gianino et al. [19]	“To assess the burden of COVID-19 in some of the EU/EEA countries”.	10 months	16 European Countries	Mortality and morbidity	Different daily and weekly reports	Observational	YLDs and YLLs	No funding received
10.	Salinas-Escudero et al. [20]	“To assess the DALYs due to COVID-19 in Mexico”.	10 moths	Mexico	Mortality and morbidity	Mexican Ministry of Health	Observational	YLDs and YLLs	Secretaría de educación, ciencia, tecnología e innovación de la
11.	Singh et al. [21]	“To put the disease impact into context and support future pandemic policy development.”	11 months	India	Mortality and morbidity	Official records or published literature	Observational	YLDs and YLLs	No funding received
12.	Jo et al. [22]	“This study aimed to calculate the burden of disease due to COVID-19 in Korea”.	3 months	Korea	Mortality and morbidity	Korean CDC	Observational	YLDs and YLLs	Korea Health Industry Development Institute

*YLDs* Years Life lost due to Disabilities, *YLLs* Years Life Lost due to premature deaths, *DALYs* Disability Adjusted Life Year

Four studies [9, 13, 18, 19] were conducted in Europe, while five studies [11, 12, 14, 16] had a global coverage. Six studies [9, 13, 18, 20–22) assessed the impacts of COVID-19 in an individual country (Malta, Korea, Germany, Mexico, India, and Scotland) and the remaining six studies covered more than one country (Table 1).

The included studies had a data collection duration ranging from 3 months to one and a quarter year. Eleven studies used observational study design and the DALYs were expressed in years lost per 100,000 people. Since there was no disability weighting assigned for COVID-19, all studies used the disability weight of severe lower respiratory disease (0.133) [10].

Four studies solely collected mortality data [12, 14–16], with the remaining eight studies using mortality and morbidity reports from health institutions and measured the impact of COVID-19 on both the quality of life (YLD) and the length of life (YLL) (Table 1). Four of the reviewed studies [12, 14, 16] assessed the years life lost due to COVID-19 related to premature deaths (length of life) only, without including the years life lost due to disability (illness, distress, and suffering) (Table 1). As a result, the health quality outcome (YLDs) from COVID-19 was not indicated and the DALYs of COVID-19 for these studies were computed from the years life lost (YLLs) due to mortality only.

### Risk of bias in studies

Based on the JBI critical appraisal tool for cross-sectional studies, only two studies [22, 23] measured the years life lost due to disability using all components of YLDs (pre-recovery time, pre-mortality time, and long-term consequence time). Four studies [12, 14, 16] ignored the YLDs from the DALYs calculation and these studies did not follow the standard criteria of DALYs [10] health impact assessment. Only one study [13] considered confounding factors like gender, age, pre-existing health conditions and economic circumstances of COVID-19 patients and used statistical strategies to account for these factors (Table 2). Six studies [14–16, 18, 20, 21] considered confounding factors, but did not use any statistical strategies to deal with them, while five studies [9, 11, 12, 19, 22] did not consider confounding factors at all (Table 2).

**Table 2:** Included studies’ risk of bias assessment results

Studies	Assessment questions/checklist								Overall appraisal
	Q1. Were the criteria for inclusion in the sample clearly defined?	Q2. Were the study subjects and the setting described in detail?	Q3. Was the exposure measured in a valid and reliable way?	Q4. Were objective, standard criteria used for measurement of the condition?	Q5. Were confounding factors identified?	Q6. Were strategies to deal with confounding factors stated?	Q7. Were the outcomes measured in a valid and reliable way?	Q8. Was appropriate statistical analysis used?	
Cuschieri et al. [9]	Yes	Yes	No	Yes	No	No	Yes	Yes	IN
Fan et al. [11]	Yes	Yes	No	Yes	No	No	Yes	Yes	IN
Oh et al. [12]	Yes	Yes	No	No	No	No	No	Yes	IN
Wyper et al. [13]	Yes	Yes	No	Yes	Yes	Yes	Yes	Yes	IN
Islam et al. [14]	Yes	Yes	No	No	Yes	No	No	Yes	IN
Pifarre et al. [15]	Yes	Yes	No	No	Yes	No	No	Yes	IN
Ugarte et al. [16]	Yes	Yes	No	No	Yes	No	No	Yes	IN
Rommel et al. [18]	Yes	Yes	No	Yes	Yes	No	Yes	Yes	IN
Gianino et al. [19]	Yes	Yes	No	Yes	No	No	Yes	Yes	IN
Salinas-Escudero et al. [20]	Yes	Yes	No	Yes	Yes	No	Yes	Yes	IN
Singh et al. [21]	Yes	Yes	Yes	Yes	Yes	No	Yes	Yes	IN
Jo et al. [22]	Yes	Yes	Yes	Yes	No	No	Yes	Yes	IN

Answer alternatives for each item are: Yes, No, Unclear, Not Applicable (N/A)

Overall appraisal alternatives are include (IN), exclude (EX), seek further information (SFI), Q stands for “question”

### Result synthesis of individual studies

All the studies used incidence rate as an epidemiological case rate measure and the data were collected using daily COVID-19 morbidity and/or mortality reports. In each study, the years life lost due to COVID-19 related mortality was higher than years life lost due to COVID-19 related disabilities (times from the onset of COVID-19 to recovery, from diseases occurrence to mortality and the long-term consequences of COVID-19). Four of the studies [9, 13, 19, 21] used the 2019 Global Burden of Diseases (GBD) standard life expectancy table, while others used the GBD-2017 [11, 12, 15, 16, 18], the GBD-2018 [22], and the GBD-2010 [14, 20] (Table 3).

**Table 3:** The COVID-19 health impact assessment results of individual reviewed studies

No.	Studies	Case rate measures	Standard life expectancy used	Result of individual studies		
				YLDs	YLLs	DALYs
1.	Cuschieri et al. [9]	Incidence	GBD-2019	250	5, 229	5, 479
2.	Fan et al. [11]	Incidence	GBD-2017	4, 399	27, 531	31,930,000
3.	Oh et al. [12]	Incidence	GBD-2017	0	4,072,325	4,072,325
4.	Wyper et al. [13]	Incidence	GBD-2019	2, 048	100,333	102, 381
5.	Islam et al. [14]	Incidence	GBD-2010	0	28,100,000	28,100,000
6.	Pifarre et al. [15]	Incidence	GBD-2017	0	20,507,514	20,507,514
7.	Ugarte et al. [16]	Incidence	GBD-2017	0	4,210,657	4,210,657
8.	Rommel et al. [18]	Incidence	GBD-2017	2033	303,608	305,641
9.	Gianino et al. [19]	Incidence	GBD-2019	17,105	835,685	852,790
10.	Salinas-Escudero et al. [20]	Incidence	GBD-2010	39,202	2,126,222	2,165,424
11.	Singh et al. [21]	Incidence	GBD-2019	112, 803	13,987,619	14,100,422
12.	Jo et al. [22]	Incidence	GBD-2018	260.3	2270.7	2531

*GBD* Global burden of diseases, *YLDs* Years Life lost due to Disabilities, *YLLs* Years Life Lost due to premature deaths, *DALYs* Disability Adjusted Life Years

Wyper et al. [13] calculated all YLDs, YLLs, and DALYs in the form of minimum and maximum ranges and we reported the average figures calculated from the lower and upper bound of each metrics. Less than half (42%) of the studies [14–16, 18, 22] reported that males were more affected by COVID-19 than females, while also noting that older people were more affected than children and adults.

### The years life lost (YLD) calculations variations among included studies

YLLs due to COVID-19 were assessed using different life expectancy standard tables among the reviewed studies. The difference of life expectancy in different life expectancy standard tables can be a source of bias in calculating the actual burden of diseases. The YLDs estimation mechanisms in the reviewed studies were not uniform (heterogeneous) among the studies. Except for the studies [12, 14–16] conducted on the YLLs due to COVID-19, the remaining eight studies considered the pre-recovery years life lost (the illness time from the onset of diseases to recovery/discharge from hospital). The number of days considered for pre-recovery YLDs calculation was variable among studies. Some studies [9, 11] use 14 days pre-recovery disability time and one study [21] used 7 days for moderate cases and 18 days for severe cases (Table 4). Jo et al. [22] used 28.4 days pre-recovery disability time and Rommel et al. [18] used 5 days of pre-recovery disability time. The remaining studies [13, 19, 20] had considered a different pre-recovery time for each case and it was not possible for us to summarise, and we therefore classified it as not specified.

**Table 4:** The COVID-19 life quality impact assessment considerations of each study

No.	Studies	Considerations for YLDs calculation/COVID-19’s impact on the quality of life		
		Pre-recovery disability time	Pre-death disability time	Long-term consequence time
1.	Cuschieri et al. [9]	Yes, 14 days	No	Yes, 28 days
2.	Fan et al. [11]	Yes, 14 days	No	No
3.	Oh et al. [12]	No	No	No
4.	Wyper et al. [13]	Yes, number of days not specified	No	Yes, 28 days
5.	Islam et al. [14]	No	No	No
6.	Pifarre et al. [15]	No	No	No
7.	Ugarte et al. [16]	No	No	No
8.	Rommel et al. [18]	Yes, 5 days	No	No
9.	Gianino et al. [19]	Yes, number of days not specified	No	No
10.	Salinas-Escudero et al. [20]	Yes, number of days not specified	Yes, number of days not specified	No
11.	Singh et al. [21]	Yes, 7 days for moderate and 18 days for sever	Yes, number of days not specified	Yes, 60 days
12.	Jo et al. [22]	Yes, 28.4 days	No	Yes, 14 days

Except for two studies [20, 21], the disability time from the onset of diseases to death was not considered in DALYs calculation. In the same way, only four studies [9, 13, 21, 22] considered the long-term consequence time in YLDs estimation. In two studies [9, 13], 28 days were considered as a disability time for long-term consequences of COVID-19 (Table 4). More than these two studies, Singh et al. [21] had considered 60 days of long-term consequences of COVID-19. The smallest long-term consequence disability time (14 days) was used by Jo et al. [22]. In all studies the presence or absence of comorbidity was not assessed.

### Certainty of evidence

Based on the GRADE handbook, the certainty assessment of each study and the overall certainty are indicated in Table 5. The certainty was assessed using the two COVID-19 health outcomes (life quality and life length). The COVID-19’s life quality impacts were assessed in three disability time frames (pre-recovery disability time, pre-death disability time, and long-term consequence disability time). Each study was assessed based on its outcomes and judged based on the GRADE scoring modalities (*high, moderate, low, and very low*). The study with one “very low” and three “high” scores were graded as “moderate” and the studies that has two “very low” and two “high” scores were graded as “low,” whereas the study with three “very lows” and one high were graded as “very low.” The study with four “highs” was graded as “high” (Table 5). Evidence on the morbidity (life quality) outcomes was judged as low with one “high” and two “very low” scores. The overall certainty of outcomes was judged as “moderate,” with one “high” (life length evidence certainty) and one “low” (life quality evidence certainty). The certainty assessment indicated that there was high certainty of evidences for life length (mortality) assessments and low certainty for life quality (morbidity) assessments.

**Table 5:** Certainty of evidence assessment result based on GRADE handbook

No.1.	Studies	Inclusion of life quality metrics				Overall certainty of each individual study
		Length of life (YLL)	Quality of life (YLD)			
			*Pre-recovery disability time*	*Pre-death disability time*	*Long-term consequence time*	
1.	Cuschieri et al. [9]	High	High	Very low	High	Moderate
2.	Fan et al. [11]	High	High	Very low	Very low	Low
3.	Oh et al. [12]	High	Very low	Very low	Very low	Very low
4.	Wyper et al. [13]	High	High	Very low	High	Moderate
5.	Islam et al. [14]	High	Very low	Very low	Very low	Very low
6.	Pifarre et al. [15]	High	Very low	Very low	Very low	Very low
7.	Ugarte et al. [16]	High	Very low	Very low	Very low	Very low
8.	Rommel et al. [18]	High	High	Very low	Very low	Low
9.	Gianino et al. [19]	High	High	Very low	Very low	Low
10.	Salinas-Escudero et al. [20]	High	High	High	Very low	Moderate
11.	Singh et al. [21]	High	High	High	High	High
12.	Jo et al. [22]	High	High	Very low	High	Moderate
*Overall certainty of YLD subsets*			*Moderate*	*Very low*	*Very low*	
*Overall certainty of YLL and YLD*		*High*	*Low*			
**Overall certainty of outcomes (DALYs)**		**Moderate**				

The studies that did not include the life quality metrics were graded as “*very low”* certainty and the studies that includes the life quality metrics were graded as “*high”* certainty

## Discussion

The aim of this review was to summarise the findings of studies on the impact of COVID-19 on health using the DALYs metrics as the health impact assessment technique. This review can be taken as a foundation for future pandemic preparedness and emergency response. The reviewed studies indicated that the COVID-19 pandemic is causing crises in both the quality and length of life. Based on the compiled information identified from the included studies, it is recommended that health policymakers and decision-makers prioritise future intervention options, either intervening in the health quality improvement or life length increment. Specific findings are discussed in greater detail in the following section.

### The impact of COVID-19 on the quality of life (YLDs)

The impact of diseases on the quality of life is measured using the number of years life lost due to disability (YLDs) [10, 18, 22, 23]. For the assessment of YLDs due to COVID-19, three important disability times should be considered:

1. the number of healthy years of life lost from the onset of the disease to recovery (pre-recovery disability time);
2. the number of healthy years of life lost from recovery/discharge from hospital to complete recovery of sequelae (long-term consequences disability time); and
3. the number of healthy years of life lost from the onset of the diseases to death (pre-mortality disability time) [13, 21].

To effectively assess the impact of COVID-19, all these disability times should be considered for YLDs estimation [13].

From all the included studies, four [12, 14–16] did not consider YLDs in DALYs calculation while the remaining eight had assessed the impact of COVID-19 due to both disability and mortality. In each of the findings, the contribution of YLDs to the overall impact (DALYs) of COVID-19 is small (Table 3). This could be due to a number of reasons including:

- The morbidity time of COVID-19 is noticeably shorter when compared to the years lost due to mortality from COVID-19. For instance, the acute phase of COVID-19 was estimated as 14 days (0.038 years) [9, 11], 5 days [18], 28.4 days [22], and 18.1 days [24]. The long-term consequence time of COVID-19 was estimated 14 days/0.038 years [22], 28 days/0.077 years [9], and 60 days/0.16 years [21].
- The pre-mortality times [9, 11, 13, 18, 19, 22] and long-term consequences [11, 18, 20] were not considered by most of the reviewed studies and YLDs were totally omitted by four studies [12, 14–16].

Due to these reasons, the share percentage of YLDs in DALYs was less than YLLs in each study. Conversely, the YLDs due to communicable diseases other than COVID-19 was 71% of the total DALYs, with the remaining 29% contributed by YLLs [25]. In addition, the proportion of YLDs and YLLs are depending on the fatality and morbidity rate of a disease. The share percentage of YLDs to YLLs in studies that calculated both YLDs and YLLs as health burden metrics were 4.56% [9], 13.78% [11], 2.01% [19], 10.28% [22], 0.67% [18], 1.81% [20], 0.8% [21], and 2% [13] of the DALYs. Mortality is more sensitive than morbidity, as deaths have a greater chance of being officially reported than morbidity. In addition, many mild and some severe cases might not be reported due to remoteness from the health facilities or a lack of confirmatory diagnosis in rural areas. However, over-reporting due to false-positive cases not supported by appropriate laboratory diagnosis may also inflate YLDs. As a result, the studies on the COVID-19 health impact due to disability were potentially exposed to reporting biases.

### The impact of COVID-19 on the length of life (YLLs)

The life length impact of a disease is measured using the years of life lost due to premature death (YLLs) [26]. According to the revised and simplified WHO global health estimate (GHE) guideline, the YLL for a particular case is calculated using the life expectancy at the time of death multiplied by the disability weight (1) [10]. Since the disability weight for death is 1, the YLL of a population due to a specific cause of a disease is equal to the life expectancy at the age of death multiplied by the number of deaths at that age. Unlike the YLDs, there is no variability in YLLs due to the disability weight of diseases [27].

The number of healthy years of life lost due to mortality has a substantial contribution to the general negative health impact of COVID-19. YLL covers 95.44% [9], 86.22% [11], 97.99% [19], 89.72% [22], 99.33% [18], 98.19% [20], 99.2% [21], 98% [13] and 100% [12, 14–16] of the DALYs found in the respective studies. Complicating this data is the fact that a substantial number of individuals who died from COVID-19 might not have been included because they lived in a remote area where deaths are not easily or consistently registered. Over-reporting due to co-mortality is also expected. For instance, the person who died primarily as the result of another disease, but who was also positive for COVID-19, may have been reported as a death due to COVID-19. For example, during the first wave of the COVID-19 pandemic, it was noted that some people in Scotland who died with dementias or circulatory causes were reported as mortality due to COVID-19 [13]. Nonetheless, the substantial negative health impact of COVID-19 indicates preparedness and caution are still needed for the ongoing deaths due to emerging COVID-19 variants and COVID-19 waves. Since it is difficult to quantify under- or over-recordings of deaths, it is acknowledged that the global health burden of COVID-19 is likely to be different to the finding of the reviewed studies, but this data still provides some insight into the probable issues.

### The disability adjusted life years due to COVID-19

The impact of COVID-19 on both the quality and length of life is measured using DALYs, which is the sum of YLD and YLL [10, 28, 29]. The number of healthy years of life lost due to a specific cause of a disease is equivalent to healthy and productive life years that were diminished by that specific cause of disease. In GBD guidelines, the value of life was dependent on the age of the patient or when they died. For example, older age groups are allocated lesser values than the productive age groups (age weighting) [30], and the years lost towards the life expectancy of a person are less valued (social discounting) [31]. The age group from 9 to 54 years were considered as more valuable than younger and older age groups [30]. However, in the simplified and revised DALYs calculation, there is no age weighting and no social discounting, and DALYs are calculated based on prevalence rate, but not incidence rate [10]. This change is helpful to avoid the complexity of diseases impact assessment [10, 32], however it does mean that studies included in this systematic review sometimes used slightly different assessment criteria. Not surprisingly therefore, there were differences in the assessed health burden of COVID-19 amongst the studies. Other factors that may explain the heterogeneity of results includes the differences in determining pre-recovery disability time, assessing only YLL [12, 14–16] without considering YLD, omitting the time of illness from the onset of diseases to death (pre-death disability time), not considering the long-term consequences (time lost due to tired, fatigue, and whole-body pain), using variable DALYs calculation guidelines, and the variations of disability time for long-term consequences [9, 13, 21, 22].

According to Fan et al. [11], the 2020–2021 global health burden of COVID-19 (31,930,000 DALYs) is larger than the annual DALYs of other infectious diseases (Malaria (42, 280), Tuberculosis (36), Lymphatic filariasis (5, 644), Leishmaniasis (2357), Schistosomiasis (1760), Trypanosomiasis (1598), Rabies (1160), Onchocerciasis (987), Chagas (649), Dengue (653), and Leprosy (177)) [33]. COVID-19 affected the life length of males and older people more than females, children, and adults [9, 15], although it is noted that patients aged 60 to 65 years were more affected than other age groups in one study [16]. As a result, health regulators, impute suppliers and countries are recommended to prepare for the prevention of health crises due to the emergence of new COVID-19 variants or other inevitable future pandemics; while the need for support for the older cohort of our community is already well known, there could also be due consideration as to whether gender is a factor that should be further explored.

### Recommendations and implications

Based on the finding of this systematic review, the following recommendations and implications for health regulators, policy makers, and health input suppliers were provided.

#### Enforcing preventive and control measures

The health regulators’ enforcement of the COVID-19 prevention and control measures is naturally going to change over time. Public awareness of the importance of COVID-19 prevention measures and follow-up of its compliance is recommended to minimise the future impact on DALYs. Further, it is likely that there will be additional benefits from the application of COVID-19 prevention and control measures in the prevention of other communicable diseases.

#### Supporting vulnerable cohorts

Since COVID-19 is a common issue across all nations, it is necessary to support each other and share resources; the control of COVID-19 in one area of the world does not ensure the eradication of the disease permanently if it is still common in other countries. If we are seeking to improve DALYs, it is suggested that supporting vulnerable cohorts, both within and across countries, is vital if we are to achieve the common goal of either eradication or minimisation of COVID-19. Vaccination and applying preventive and control measures can aid in reducing the impact of the COVID-19 pandemic of DALYs [34, 35].

#### Further research

Obviously, the ongoing nature of the pandemic means that suggestions for definitive research foci are not yet appropriate as the data remains incomplete. However, the results from this initial systematic review on COVID-19 has highlighted its multi-faceted impact, and future research focussing on the disease’s progress and health impact assessments that specifically analyse the years lost due to disabilities and mortality are recommended.

### Limitations of the reviewed studies and review process

The selected studies were affected by publication, selective outcome, and selective analyses biases. Many of the studies did not report the comorbidity and the demographic effect of COVID-19. Most of the studies did not consider the pre-death and long-term consequence disability times for calculating YLDs. Studies used the old GBD guidelines instead of the revised and simplified GHE standard for DALYs estimation. In the contrary, the DALYs, YLLs and YLDs numerical figures indicate in this review are based on the reported cases and deaths of COVID-19 at the time of each study and did not include unreported cases and deaths from the same study area or other parts of the world. As a result, the COVID-19 health impact assessments shown by the reviewed studies did not indicate the actual global burden of COVID-19. Since the studies did not estimate how many the unreported cases are in each study area, we cannot discus on the unreported morbidity and mortality cases of COVID-19.

We used the title and abstract screening technique. A recent study confirmed that abstract screening misses around 13% of valuable studies [36]. Due to time constraints, we selected only full-length articles, but reports, unpublished studies, and reviews were not included. Our restriction to the English language may have led us to miss valuable studies reporting on data in other countries. In addition, due to the complexity of the COVID-19 impact assessment, some searching terminologies like YLLs and YLDs could not be included, which can be a further source of missing literature. Since majority of the reviewed studies covered more than one country, the COVID-19 health impact of one country might be reported by more than one study that led to over COVID-19 health impact estimates.

### Registration and protocol

The systematic review was registered in PROSPERO under the registrations number CRD42022324931. Before the start of the systematic review, a review protocol was prepared, agreed upon by all review authors and published in a reputable journal [17].

### Amendments of published protocol

The protocol of this systematic review [17] was published before the end of this article. Since the proposed risk of bias assessment tool (ROBIS) is more appropriate for systematic reviews than primary research articles, the risk of bias for the included studies of this systematic review was assessed using JBI cross-sectional studies critical appraisal tool.

## Data Availability

All data related to this review is included in the result section of the manuscript. If any further data is needed it can be accessible via the corresponding author on request.
